# Supplementary material for: Content-rich biological network constructed by mining PubMed abstracts
Source: BMC Bioinformatics. 2004 Oct 8;5:147. doi: 10.1186/1471-2105-5-147 (PMC528731; doi:10.1186/1471-2105-5-147)
Supplement: Additional File 2 — The original results of the above study (non-essential files are deleted to keep the file size under the limit set by BMC bioinformatics). [file 1471-2105-5-147-S2.bz2 › chilibotAdditionalFile2/dip20/379ID9927421E9792/html/NGFR_NTRK3.html]

 


 **NGFR** and **NTRK3** 
  
Found 76 abstracts in PubMed, retrieved 20.  
 

 What does Google say? 
 PDF only 
| .edu only 

---

**Interactive relationship** (e.g. stimulation, inhibition, etc)

**Neutral relationship**- By immunohistochemistry, p75NTR  [ **NGFR** ]  alone was strongly expressed in TUNEL Bcl2 keratinocytes of the regressing outer root sheath, but both p75NTR  [ **NGFR** ]  and TrkB and or TrkC  [ **NTRK3** ]  were expressed by the nonregressing TUNEL Bcl2 secondary hair germ keratinocytes.  Ref: 11023977 FASEB J, 2000

**Non-interactive relationship** (e.g. studied together, co-existance, homology, etc.)

- This is coupled to the ectopic expression of trk B and trk C  [ **NTRK3** ] , as well as to the continued expression of trk A, and the loss of expression of p75NTR  [ **NGFR** ]  receptors.  Ref: 11027413 Prostate, 2000
- NT 3 mediated death induction requires co signalling of TrkC  [ **NTRK3** ]  and p75NTR  [ **NGFR** ]  receptors.  Ref: 11236067 Rev Neurosci, 2001
- METHODS Immunocytochemistry and Western blot analyses were performed using primary antibodies raised against NTs nerve growth factor NGF, brain derived neurotrophic factor BDNF, NT 3, and NT 4 5 and NT receptors TrkA, TrkB, TrkC  [ **NTRK3** ] , and p75NTR  [ **NGFR** ] .  Ref: 11053307 Invest Ophthalmol Vis Sci, 2000
- Mouse PTPRO mRNA was observed in neuron populations expressing TrkA, the high affinity nerve growth factor receptor  [ **NGFR** ] , or TrkC  [ **NTRK3** ] , the neurotrophin 3 receptor  [ **NTRK3** ] , and immunoreactive mPTPRO and TrkC  [ **NTRK3** ]  colocalized in large dorsal root ganglia proprioceptive neurons.  Ref: 12532410 J Comp Neurol, 2003
- Our results show that myelin formation is inhibited in the absence of functional p75NTR  [ **NGFR** ]  and enhanced by blocking TrkC  [ **NTRK3** ]  activity.  Ref: 12424382 Science, 2002
- With BDNF TrkB promoting survival and NT 3 TrkC  [ **NTRK3** ]  p75NTR  [ **NGFR** ]  promoting death, CSN integrate at least three different neurotrophin receptor signals for death survival decisions.  Ref: 11236067 Rev Neurosci, 2001
- Thus p75NTR  [ **NGFR** ]  and TrkC  [ **NTRK3** ]  receptors have opposite effects on myelination.  Ref: 12424382 Science, 2002
- In order to gain insight into specific roles for TrkC  [ **NTRK3** ]  NC2 receptors during CNS neurogenesis, we compared its distribution with that of its catalytic counterparts and the p75NTR  [ **NGFR** ]  receptor in in vivo and in vitro model systems of early and late neuronal differentiation.  Ref: 10998105 Eur J Neurosci, 2000
- ... the expression of the neurotrophin, BDNF, the NT 3 receptor, TrkC  [ **NTRK3** ] , and the nonselective, low affinity neurotrophin receptor p75NTR  [ **NGFR** ] , were comparable to wild type.  Ref: 11848682 Neurobiol Dis, 2002
- Spatiotemporal expression of noncatalytic TrkC  [ **NTRK3** ]  NC2 isoform during early and late CNS neurogenesis a comparative study with TrkC  [ **NTRK3** ]  catalytic and p75NTR  [ **NGFR** ]  receptors.  Ref: 10998105 Eur J Neurosci, 2000
- Virtually all adult CSN express mRNA coding for the NT 3 receptor TrkC  [ **NTRK3** ]  and the BDNF receptor TrkB, and after axotomy, CSN also express mRNA for the common neurotrophin receptor p75NTR  [ **NGFR** ] , suggesting a role of endogenous neurotrophins for survival regulation of CSN.  Ref: 11236067 Rev Neurosci, 2001
- The specimens were analyzed immunohistochemically for neurotrophins nerve growth factor, brain derived neurotrophic factor, neurotrophin 3, neurotrophin 4 5 and their receptors TrkA, TrkB, TrkC  [ **NTRK3** ] , p75NTR  [ **NGFR** ] .  Ref: 11442674 Int J Urol, 2001
- Neurotrophin signaling is mediated by Trk family tyrosine kinases such as TrkA, TrkB and TrkC  [ **NTRK3** ] , as well as by the pan neurotrophin receptor p75NTR  [ **NGFR** ] .  Ref: 11836532 Nat Neurosci, 2002
- Although neurotrophins did not regulate p75NTR  [ **NGFR** ]  processing, the alpha and gamma secretase mediated cleavage of p75 is modulated by receptor tyrosine kinases Trks TrkA and TrkB but not TrkC  [ **NTRK3** ] .  Ref: 12843241 J Neurosci, 2003
- Retinal degeneration upregulated both p75NTR  [ **NGFR** ]  and the high affinity neurotrophin receptor TrkC  [ **NTRK3** ]  in different parts of Müller glial cells.  Ref: 10839371 Neuron, 2000
- Sections of pancreatic cancer and normal pancreatic tissue were examined by immunohistochemistry for the expression of the neurotrophins NGF, BDNF, NT 3, NT 4, and their receptors TrkA, TrkB, and TrkC  [ **NTRK3** ] , as well as the low affinity receptor, p75NTR  [ **NGFR** ] .  Ref: 11561004 J Histochem Cytochem, 2001
- During neuritogenesis of primary neocortical neurons, both TrkC  [ **NTRK3** ]  isoforms as well as p75NTR  [ **NGFR** ]  were located in axonal and dendritic processes.  Ref: 10998105 Eur J Neurosci, 2000
- We have used immunofluorescence techniques with a panel of antibodies against known neurotrophin receptors trk A, trk B, trk C  [ **NTRK3** ] , p75NTR  [ **NGFR** ]  to map the locations of these receptors in the developing neuromuscular system of mice from our neurotrophin 3 NT 3 knockout colony.  Ref: 12141445 Int J Dev Biol, 2002
